# EFFECT OF INFLUENZA VACCINE ON TWEAK LEVELS IN THE ELDERLY: IMPLICATION IN CARDIOVASCULAR PROTECTION

**DOI:** 10.1093/geroni/igz038.2392

**Published:** 2019-11-08

**Authors:** Juliette Tavenier, Min Ouyang, Huifen Li, Sean X Leng

**Affiliations:** 1 Clinical Research Centre, Copenhagen University Hospital, Hvidovre, Hvidovre, Denmark; 2 Department of Geriatrics, The Second Xiangya Hospital, Central South University, Changsha, Hunan, China; 3 Johns Hopkins University School of Medicine, Baltimore, Maryland, United States

## Abstract

Substantial evidence suggests a protective effect of annual influenza immunization on cardiovascular diseases (CVD). The inflammatory mediator TNF-related weak inducer of apoptosis (TWEAK) is thought to be involved in the pathogenesis of CVD. We previously showed that administration of a standard-dose influenza vaccine reduced circulating TWEAK levels. This study aimed to test the hypothesis that a high-dose vaccine would have greater impact on TWEAK levels than the standard-dose. Two groups of participants matched on age and sex were included in the study. One group (n=25) received standard-dose vaccine during 2008-2009 season, the other group (n=25) received high-dose vaccine during 2014-2015 season. Soluble TWEAK (sTWEAK) levels were assessed using ELISA in serum samples collected immediately before vaccination and during the 4th week after vaccination. Vaccine-induced strain specific antibody titers were measured by hemagglutination inhibition assay. The participants had a mean age 86 years and 68% were women. Our preliminary results thus far demonstrated no statistically significant change in sTWEAK levels after vaccination in either group (Wilcoxon matched-pairs signed rank test: standard-dose group: median change [interquartile range]=11.2 [-92.2–197.1] pg/mL, p=0.72; high-dose group: -24.8 [-58.9–87.3] pg/mL, p=0.70). Pre-vaccination sTWEAK levels tended to be negatively associated with age (unadjusted linear regression of log2(x) transformed TWEAK levels on age: estimate [±SE]=-4.6% [±2.7%] change in TWEAK level per year, p=0.08). We continue to evaluate more pre- and post-vaccination samples. We have also begun exploring TWEAK expression by circulating immune cells (T and B lymphocytes and monocytes) and potential impact of influenza immunization in older adults.

